# Assessment of the Wearability of Facemasks against Air Pollution in Primary School-Aged Children in London

**DOI:** 10.3390/ijerph17113935

**Published:** 2020-06-02

**Authors:** Naomi R Smart, Claire J Horwell, Trevor S Smart, Karen S Galea

**Affiliations:** 1 Bristol Medical School, First Floor, 5 Tyndall Avenue, Bristol BS8 1UD, UK; ns15067@bristol.ac.uk; 2 Institute of Hazard, Risk & Resilience, Department of Earth Sciences, Durham University, Lower Mountjoy, South Road, Durham DH1 3LE, UK; 3 UCB Pharma, Statistical Sciences and Innovation, Slough SL1 3WE, UK; trevor.smart@ucb.com; 4 Institute of Occupational Medicine (IOM), Research Avenue North, Riccarton, Edinburgh EH14 4AP, UK; karen.galea@iom-world.org

**Keywords:** facemask, children, wearability, air pollution

## Abstract

Air pollution is a major health problem and children are particularly vulnerable to the adverse effects. Facemasks are one form of protection but, to be effective, they need to filter out airborne pollutants, fit the face well and be wearable. In this pilot study, we assess the perceived wearability of three facemasks (Vogmask, TuHao and ReSpimask) marketed in the UK as being designed to protect children against exposure to air pollution. Twenty-four primary school children wore each facemask during a standardised walking and running activity. After each activity, the children were asked to rate facemask wearability in terms of parameters, such as perceived comfort, hotness, breathability and fit. At the end of the trial, the children compared and identified their preferred facemask. The main complaint about the facemasks was the children’s faces being too hot. The ReSpimask was most frequently reported as being perceived to be the hardest to breathe through. The TuHao facemask was the only adjustable strap mask assessed but was reported to be difficult to adjust. Facemasks with a nose clip were frequently rated highest for fit (TuHao and Vogmask). The patterned, cloth fabric Vogmask had significantly higher ratings for appearance and perceived fit. The results show children’s perceptions of facemasks are highly affected by the facemask’s design, hotness and perceived breathability. By making children’s facemasks more appealing, breathable, cooler and improving their fit, wearability may be improved.

## 1. Introduction

Exposure to outdoor air pollution is a major cause of disease, hospital admissions and premature death, globally, with children being one of the most susceptible populations [1,2,3,4]. In the UK, 40,000 deaths every year are linked to exposure to outdoor air pollution [1]. It has been estimated that 10% of childhood asthma hospital admissions in London are due to air pollution [5].

As the general public have become more aware of the risks of air pollution, buoyed in recent years by frequent media attention and activism, companies have capitalised on public anxiety and started to produce non-occupational facemasks (although sometimes made from industry-certified materials) designed to filter fine particulate matter and sometimes gaseous pollutants [6]. Some companies are also manufacturing smaller-sized versions of adult masks for children [7]. The COVID-19 crisis has also greatly increased demand for, and awareness of, facemasks for public use [8].

Facemasks are not considered to be a suitable primary intervention for outdoor air pollution exposure reduction for children, for several reasons. The main reason is that eliminating or reducing exposures through removing sources of emissions near where children gather (e.g., schools) is the greater aim and benefit [9,10,11]. Additionally, facemasks traditionally would not fit children correctly as they were designed for adult-sized faces [11,12] and it is unlikely that a child would keep a mask on, or wear it properly, for a prolonged period, thereby substantially reducing the efficacy of the intervention [13]. In addition, facemasks could increase the effort in breathing, due to increased breathing resistance and a reduction in the volume of air breathed, which can result in discomfort and fatigue [14].

However, reducing emissions sources is a slow process and some children may have no alternative but to be exposed to air pollution on the way to school and during other outdoor activities. Therefore, personal interventions, such as facemasks, are possible viable solutions if they are of industry standard, fit well and are worn properly (assuming there are no medical issues which would be exacerbated due to their use). Evidence must be provided, therefore, on the efficacy of these masks, their ability to fit children’s faces and their likelihood of uptake by children due to their perceived wearability. This study addresses wearability.

If masks are uncomfortable, annoying or embarrassing to wear, the motivation to wear them, or to keep them on, may be limited and they will provide inadequate protection [7,11,15,16]. There is currently limited non-occupational research into the wearability of facemasks, irrespective of whether they are certified (passing laboratory testing as being capable of filtering particulate matter, for example, capable of filtering 95% of small (0.3 µm) particles (i.e., the US N95 standard, equivalent to Filtering Face Piece 2 (FFP2) in Europe)) or not.

At present, facemasks marketed for children are usually small sized adult ones [7,17,18,19], potentially reducing their wearability, due to appearance and fit, although several manufacturers are now designing masks for children. To date, there has been one study that evaluated the safety, fit and comfort of an N95 mask designed for children [7]. Goh et al. (2019) conducted a randomised clinical crossover trial of 106 children aged 7–14 who wore the Air^+^ Smart Mask (with optional microventilator) and a control (no mask) [7]. As well as testing children’s physiological responses in different states of physical activity, and conducting fit tests, the subjects were asked to rate if they experienced breathing difficulty on a visual analogue scale. Ninety-three percent of the children perceived that they experienced no breathing difficulty and 7% perceived mild breathing difficulty. To our knowledge, this is the only assessment of comfort of facemasks for children, prior to this study, and it should be noted that the study was commissioned and funded by the manufacturer of the masks.

Galea et al. (2018) conducted a study with adults in Indonesia, testing the wearability of facemasks used as protection against volcanic ash, including N95 masks and surgical masks [20]. Results highlighted some wearability barriers to the uptake of some facemasks which included perceptions of poor fit, comfort and breathability. These results were backed up by a laboratory-based study with volunteers who wore the same masks [21]. Previous research has shown that children can have different reactions to adults concerning wearing facemasks [22]. Children do not necessarily understand the reasons for wearing a facemask: the mask may have a poor fit leading to the mask slipping or ripping; the child may also experience discomfort when wearing the mask and verbal communication can feel restricted. These factors can all contribute to children removing facemasks [22,23].

The purpose of the current pilot study was to assess perceptions of wearability of facemasks marketed in the UK to protect children from inhaling air pollution, based on criteria such as perceptions of comfort, hotness, fit, and the ease of breathing. The wearability of three facemasks were assessed: Vogmask, TuHao and ReSpimask [17,18,24]. Table 1 outlines key features of these masks. These facemasks were selected because of their marketed N95 (or greater) filtration capacity, ease of purchase on-line by UK consumers, stock availability and, importantly, they were being sold as facemasks for children. The Vogmask and ReSpimask used in the study were donated free of charge by the manufacturers. All three masks state that they are made of N95/N99-quality materials (two with evidence of quality testing; Table 1), which was important because any facemasks without such guarantee would likely have low filtering efficiencies and so were unsuitable for inclusion for ethical reasons. Additionally, we wanted to ensure that filtration capacity was not a factor influencing the children’s perceptions of wearability.

## 2. Materials and Methods 

### 2.1. Ethical Approval and Exclusion Criteria

Ethical approval was given by the Ethics Board, Faculty of Health Science Student Research Ethics Committee (HSSREC), University of Bristol. Written informed consent for the children to participate in the study was provided by the children’s parents/guardians, as well as verbal assent from the children on the day. As a precautionary measure, children with underlying respiratory conditions, cardiovascular problems and claustrophobia were excluded from participating in the study.

### 2.2. Participant Recruitment 

The study population were girls and boys, aged 8–11 years, from a primary school in London, United Kingdom (UK). All children in this age range at the school were given a participant information leaflet and consent form to take home to their parents/guardians. From the children who returned informed parental consent, 24 were randomly selected as the study sample and assented to participate. The sample size of 24 allowed any key issues of wearability to be identified through analysis of the results across the children and any major differences among the mask types to be identified through the intra-child comparisons of the mask types. The children were randomly assigned participant identification numbers 1–24, with only the primary researcher knowing which child was linked to which identification number. Each child completed the study in full. 

### 2.3. Experimental Set-Up 

The study took place between 5 and 7 July 2019. On each day, every child was allocated one facemask and, over the three days, each child wore each facemask type once, being randomly assigned to one of six sequences of facemask which ensured that each facemask was tested once by each child. Randomisation was performed using a random number generator, with each child randomised to one of the six sequences given.

On the first day of the study, a short, qualitative questionnaire was administered to each child. Children were asked what they think of when they hear the term ‘air pollution’, what they think the causes of air pollution are and whether they think there are any effects to health from breathing air pollution. Following this (and on each day of facemask testing), the facemask being tested by that child on that day was donned and the child was asked to carry out two activities: a gentle walk for three minutes, followed by three minutes of running. These activities took place after break time each day, in the school playground. At the end of each activity the children were individually asked to rate their perceptions of comfort, hotness, breathability and fit when wearing the facemask. using a five-point Likert scale. The scale used a range of facial expressions (a visual analogue scale) to help the children understand the process and provide answers more easily. If the children took their facemask off during the walk, they were replaced prior to the run, giving the opportunity to evaluate the masks’ wearability in different situations. Children were also asked if they felt embarrassed when wearing the facemask, if they liked how the facemask looked and whether they removed the facemask during the activities. The children were then asked if and when they would wear the mask again, as open questions.

After testing their final facemask, a post-activity questionnaire was administered where the children were asked to rank the three facemasks in terms of their perceptions of the comfort, ease of breathing, and the facemask they would most likely want to wear again.

Where possible, different sizes of the facemasks had been purchased/donated and these were fitted to each child by the researchers (without accurately measuring the child’s face), prior to the activity to ensure that the most appropriate size was provided. The children were encouraged to wear their facemask for the entire activity, but were made aware that they could take the facemask off at any point, if they found them uncomfortable, too hot, felt their breathing was restricted or the facemask restricted their activity. Researchers observed the children whilst walking and running, recording any observations regarding the facemask, e.g., fiddling with the facemask, the facemasks slipping off the child’s face or being removed, as well as helping reposition masks if they moved out of place. At the end of the study, the children kept the masks they had worn.

### 2.4. Data Analysis

The data of all the children were included in the analysis for each day, whether the child kept their facemask on or not, to remove potential bias. If those participants who removed the mask were excluded, then this could give an over optimistic assessment of the wearability of the mask. Each child wore each mask for sufficient time for them to assess wearability.

The facial expression Likert scale was converted to numerical values for data analysis (−2 to 2). Likewise, the scale for the embarrassment question was coded to numerical values (−2 to 2). Hence, for all these scores, a positive score reflected the child being positive in their response, a zero score is neutral and a negative score indicates a negative response.

A mixed model with child as random effect and facemask, day, and gender as fixed effects, with age as a covariate, was used to see how acceptable the facemask types were to wear and for comparison among the masks. The mean Likert ratings for each mask were used to compare among masks. Chi-squared tests were used to compare the ranking of the masks, whether the masks were removed and whether the children would wear them again. The level of significance used is a two-sided *p*-value < 0.05.

The analyses were completed in IBM SPSS 24 Statistics, Armonik, NY, USA [25].

## 3. Results

### 3.1. Participant Characteristics 

The characteristics of the 24 children are summarised in Table 2. All 24 participants completed the study in full. The results are reported for the whole study population, rather than by gender and age, due to the small number of participants within each of these categories.

### 3.2. Pre-Activity Questionnaire—Perceptions of Air Pollution 

All participants perceived air pollution as harmful for humans or the environment (e.g., ‘damages plants’). Twenty-two children (92%) believed it was bad for health, six children (25%) specifically stated that it damaged your lungs, and four children (17%) said it caused asthma and lung diseases.

All children had ideas about where air pollution came from. Fourteen children (58%) stated cars were a cause. Other modes of transport and factories were the next most frequent responses, with 11 children (46%) and nine children (38%) respectively mentioning these factors.

### 3.3. Positive and Negative Mask Ratings 

The aspect of wearability the children rated highest for all facemasks was lack of embarrassment. They were not embarrassed wearing any of the facemasks (mean = 1.5, *p* < 0.001), Table 3. The wearability criterion with the lowest score for all three masks was hotness whilst running (mean = −0.61, *p* < 0.001), Table 3.

### 3.4. Comfort

There was a significant difference in the level of comfort of the masks whilst walking (*p* = 0.013) and running (*p* = 0.003), with the children finding it more comfortable when walking. The Vogmask was given the highest mean score for comfort whilst walking (1.3) and running (0.8), Table 3, and was ranked most comfortable of the three masks tested, by 67% of the children (χ^2^ = 18.9, *p* < 0.001), Table 4. ReSpimask was consistently the lowest rated and was ranked the least comfortable by 67% (χ^2^ = 19.5, *p* < 0.001) of the children.

### 3.5. Hotness

All the mean scores for hotness during running were negative, Table 3. There was no evidence of any differences among the three facemasks in the ratings for hotness whilst walking and running (walking: *p* = 0.257; running: *p* = 0.876).

### 3.6. Breathability

There was a significant difference between the perceived breathability of the different facemasks during the running activity, only (*p* = 0.028), Table 3. TuHao and ReSpimask had similar numbers of participants ranking them as most breathable (46% and 42%), but Vogmask had significantly fewer at 12% (χ^2^ = 7.13, *p* = 0.028), Table 4.

### 3.7. Fit 

There was a highly significant difference in perception of fit among the facemasks (*p* < 0.001), Table 3. Vogmask had the highest mean score (1.5), and ReSpimask had the lowest mean score for fit (0.1). Vogmask also had a higher average rating than TuHao and ReSpimask (difference = 0.54, *p* = 0.032 and difference = 1.50, *p* < 0.001, respectively); TuHao had a higher average rating than ReSpimask (difference = 0.96, *p* < 0.001).

### 3.8. Embarrassment 

There was no significant difference among the facemasks for ratings of embarrassment (*p* = 0.203), Table 3. Most children were not embarrassed at all to wear any of the three masks (mean scores: 1.3, 1.5, 1.7 for ReSpimask, TuHao and Vogmask, respectively).

### 3.9. Appearance 

There was a highly significant difference between perceptions of the different facemasks’ appearance (*p* < 0.001), Table 3. Vogmask had the highest facemask appearance mean rating (1.6) and ReSpimask had the lowest (−0.4). Vogmask had a higher rating than TuHao and ReSpimask (difference = 0.83, *p* = 0.005 and difference = 2.00, *p* < 0.001, respectively); TuHao had a higher rating than ReSpimask (difference = 1.17, *p* < 0.001).

After being asked to rate the facemasks’ appearance, the children were invited to explain their answer. Vogmask had the highest proportion of positive comments (85%), and the lowest proportion of negative comments about its appearance (11%). Fifteen children (63%), said they liked the design of Vogmask. ReSpimask had the lowest proportion of positive comments (15%), and the highest proportion of negative comments (77%) about its appearance; for example, many children said the ReSpimask looked like a nappy/diaper.

### 3.10. Removing the Mask During Activities

Nine children (38%) removed the Vogmask and TuHao facemasks during the activities, whereas 15 children (63%) removed the ReSpimask during the activities. This was not statistically significant (χ^2^ = 4.03, *p* = 0.133). Facemasks were most often removed during running, Table 5. For 16 children (67%), the ReSpimask slipped off and had to be put back on during the activities. For five children (21%), the ReSpimask ripped and they were asked to take the mask off. These children continued walking without wearing a mask, to enable supervision of all the children.

The main reason given why the children removed their facemasks was because they got too hot (Vogmask: n = 5, 21%; TuHao: n = 8, 33%; ReSpimask: n = 4, 17%). This is consistent with the results for rating the hotness during walking and running, Table 3. Several children perceived that it became hard to breathe so they took their facemask off: Vogmask: n = 3, 17%; TuHao: n = 2, 8%; ReSpimask: n = 7, 29%.

### 3.11. Future Use 

The children were asked, after wearing each mask, whether they would wear it again. Ten children (42%) said they would for the Vogmask and TuHao facemasks, but only three (12%) said they would for the ReSpimask. ReSpimask had the highest number of children saying they would not wear it again (n = 13, 54%) (χ^2^ = 12.6, *p* = 0.002).

At the end of the experiment, when the children compared the three masks, they were asked again which mask they were most and least likely to wear. At this point, the most popular mask to wear again was Vogmask (67%, χ^2^ = 18.4, *p* < 0.001, Table 4).

The most common reason given for potentially wearing a mask in the future was related to air quality (children would wear a mask when in areas of high pollution). Several described wanting to wear the mask only in cool weather because the masks were too hot, consistent again with the results for rating the hotness during walking and running, Table 3.

Finally, the children were asked for any comments about the facemasks they had worn. The most common comment that five children (21%) made was facemasks for children need a wire in the nose and the chin to improve fit. Four children (16%) said there should be more sizes available.

## 4. Discussion

This pilot study aimed to assess the wearability of three facemasks marketed in the UK to protect children from inhaling air pollution, based on criteria such as perceived comfort, fit, and ease of breathing.

The main complaint about the facemasks was the children’s faces becoming too hot, particularly during the running activity. The days of the study were warmer than the seasonal average London temperatures (mean = 23 °C) [26], potentially contributing to the hotness and removal of masks. However, if these facemasks are designed for worldwide use, in hotter climates than London, the hotness of the facemask is likely to be an even greater wearability issue.

In this study facemasks were rated as being hotter during running—when respiratory rate increases more heat is produced—therefore, the wearability of facemasks is reduced during physical activity. Therefore, hotness is an important factor to be considered when designing facemasks for children [7].

A perception of difficulty in breathing was a factor causing the children to remove facemasks. Previous studies have also found that perceptions of increased breathing resistance reduces the wearability of facemasks in adults [20,27,28]. In this study, for all masks, breathability was one of the lowest rated wearability criteria (i.e., the children perceived the masks to negatively affect their ability to breathe). The ReSpimask was described most frequently as the hardest mask to breathe through, with the children being surprised by the mask coming into their mouth when they breathed in. This is likely to be due to the thin, delicate mask material. The perceived breathability of TuHao and Vogmask were also rated low, with the children commenting that their thick material reduced breathability.

The perception of fit was a significant factor affecting the perceived wearability of the facemasks, as has also been seen when assessing the wearability of adult facemasks [20,28]. A good fitting mask is also essential to provide adequate protection [7,15]. The masks tested in the adult wearability studies often had adjustable components to improve fit [20,21]. The TuHao mask was the only adjustable mask in this study, with adjustable ear loops, however, they were found to be difficult to adjust. An adjustable head strap that can be purchased separately for the Vogmask was not evaluated in the current study and it is possible that use of this this may have led to an improved fit. Masks with nose clips were frequently rated the highest for perceptions of fit (TuHao and Vogmask). The children thought the nose clip was essential and proposed having an additional clip for the chin to improve fit. ReSpimask did not have a nose clip and both sizes frequently slipped off the children’s faces, ripped or had to be constantly readjusted. Correspondingly, ReSpimask was rated lowest for fit. The Small+ ReSpimask designed for 5–10 years old was found to be too large for even the 11-year-old children in the study, therefore, it would likely not protect the children adequately from air pollution.

The appearance of the facemasks was an important factor for the children when deciding if they would wear the mask again. The Vogmask, had considerably higher ratings for appearance, compared with the TuHao and ReSpimask. Vogmask was the only mask that was not disposable nor white; instead it had a patterned front, and has a cloth exterior, and was rated as the most favourable mask overall. This study suggests that, for children, the appearance of the mask was a highly important aspect of its wearability. 

Appearance of facemasks is not necessarily deemed as important amongst adults as it is for children where, for adults, the need for comfort and the perception of effectiveness significantly affects the wearability of masks [20,21,28]. Covey et al. (2020), conducted a study exploring the perceptions of using protective facemasks against volcanic ash exposure in the population living near Sakurajima volcano, Japan [28]. They found that ease of use, breathability and comfort were the most important factors for the community. Fashion was deemed the least important factor, although those that did think it important were more likely to be younger people, women or those living in urban locations. This suggests appearance is an important factor to consider in designing wearable facemasks for both children and young adults. However, the children found none of the masks to be embarrassing to wear and this also concurs with the results in people aged 13+ in Japan, in Covey et al. (2020), a result which is somewhat unexpected since mask wearing is not part of British culture. 

The perceived acceptability of the facemasks by the children could have been influenced by social desirability bias, where people tend to give an answer which they deem to present them in a more positive light than their true answer [29,30], because inclusion in the study could have been regarded favourably by the children. We did not directly assess the potential impact of this bias on the participants’ views on the wearability of the three assessed masks. However, given the study design, particularly the random allocation of the masks to participants across the three days and the encouragement of use of free text answers to support their answers, we consider any potential social desirability would not have led to children to respond more positively (or negatively) to one mask in preference to the others assessed. However, social desirability bias is a possible explanation for the lack of reported embarrassment whilst wearing the facemasks.

Due to limited time and resources in this pilot study, only three masks were assessed, for a short period of time, the sample size was small and children’s face size and shape were not measured. Therefore, the results may not be generalizable to all children, all face masks that may be marketed for use by children, or the durations for which they may be worn. This study also only focused on the wearability of facemasks; other factors that impact the usage of facemasks were not considered, for example, cost, storage and cleaning (of reusable masks). Children with underlying respiratory conditions were excluded from this study for safety and ethical reasons, however, these children are both at a higher risk from the health impacts of air pollution and are likely to have lower acceptability of facemasks because of perceived breathing restrictions. It will be important for future research to address this. The results of this study may also have been affected by the researchers’ presence influencing the children’s behaviour (the Hawthorne effect) [31], but this does not negate the study findings. Despite these limitations, the results are statistically significant and provide new insight into the wearability of children’s facemasks. All 24 children attended the same primary school and were not tested independently (all carried out the activity at the same time), but were independently asked the questionnaires. The study was designed to assess wearability of children’s facemasks over a short time period, so we are unable to predict how the wearability of masks may differ if worn for a longer period, for example, the time taken to walk or cycle to school. It is therefore important that more studies are undertaken to assess the wearability of children’s facemasks with younger children and teenagers, and over extended periods, typical of the likely duration that facemasks may be worn by this target population. This study highlights the need for further research to assess the wearability of other facemasks designed for children.

## 5. Conclusions

If there are no other solutions available during a severe air pollution episode, then facemasks may be worn by children and need to be wearable. It is important that facemask producers and governments are aware of the barriers to children wearing facemasks and how these may differ from adults. In this study, children’s perceptions of masks were highly affected by their design, hotness and perceived breathability. A patterned, non-disposable mask was preferred. By making the children’s facemasks more appealing, breathable and cooler, their wearability would likely increase. It is important to have masks available for children that fit well and are effective, in order to provide adequate protection.

## Figures and Tables

**Table 1:** Characteristics of the three masks tested in the wearability study.

Mask	Image	Sizes Used	Protection Provided (As Stated by Manufacturers)	Nose Clip?	Adjustable Straps?	Exhalation Valve?	Reusable?	Unit Price ^1^
The Tree Premier Vogmask VMCV	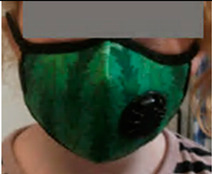	Small (youth under 10 years) Medium (10 years old to petite adult)	Protection from PM_2.5_ airborne particles. N95 rating against 0.3 µm airborne particles, filtering >99.9% of airborne bacteria and viruses, validated by Nelson Laboratories [18]	Yes	No. Adjustable Vogmask Head Strap Accessory can be purchased separately	Yes	Yes	£26.00
TuHao Kids Disposable Face Mask	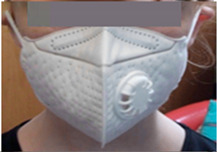	One child size	N95 filtration technology. PM_2.5_ dust mask-anti-pollution for outdoor safety multi-layer protection	Yes	Yes. Adjustable ear loops—a toggle at the back of the ear to improve fit, pulled to make the ear loop shorter	Yes	No	£0.40
Junior Disposable ReSpimask, Respilon	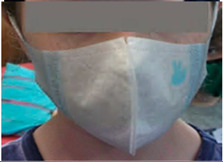	Small (2.5–4 years) Small+ (5–10 years)	PM_2.5_ particulate filter, N99 protection, validated by Nelson Laboratories and EMPA [17]	No	No	No	No	£1.88

^1^ Recommended retail price at the time of the study being undertaken in 2019.

**Table 2:** Description of participants.

Description	N	%
Gender		
Male	13	54
Female	11	46
**Age at time of recruitment (years)**		
8	6	25
9	5	21
10	9	37
11	4	17

**Table 3:** Mean (standard deviation) scores for each facemask and wearability criteria.

Wearability Criteria	Facemask Type: Mean (Standard Deviation)			*p* Value
	Vogmask	TuHao	ReSpimask	
Comfort whilst walking ^1^	1.3 (1.0)	1.0 (0.8)	0.6 (1.0)	0.013
Comfort whilst running ^1^	0.8 (1.2)	0.3 (1.3)	−0.3 (1.1)	0.003
Hotness whilst walking ^2^	−0.2 (1.1)	−0.1 (1.0)	0.3 (1.2)	0.257
Hotness whilst running ^2^	−0.7 (1.3)	−0.5 (1.1)	−0.6 (1.1)	0.876
Breathability whilst walking ^3^	1.3 (0.7)	0.8 (1.2)	1.0 (1.0)	0.206
Breathability whilst running ^3^	0.4 (1.2)	0.2 (1.3)	−0.5 (1.2)	0.028
Fit ^4^	1.5 (0.6)	1.0 (0.9)	0.1 (1.0)	<0.001
Embarrassment ^5^	1.7 (0.5)	1.5 (0.9)	1.3 (1.1)	0.203
Appearance ^6^	1.6 (0.7)	0.8 (1.1)	−0.4 (1.2)	<0.001

^1^ A positive score for comfort means the child felt the mask was comfortable to wear. ^2^ A positive score for hotness means the child didn’t feel the mask made their face too hot. ^3^ A positive score for breathability means the child felt they could breathe easily through the mask. ^4^ A positive score for fit means the child thought the mask fitted them well. ^5^ A positive score for embarrassment means the child was not embarrassed to wear the mask. ^6^ A positive score for appearance means the child liked what the mask looked like.

**Table 4:** Percentage of children who ranked the mask best for each category.

Category	% Children Who Ranked the Mask Best			
	Vogmask	TuHao	ReSpimask	χ^2^ (*p* Value)
Which was the most comfortable mask?	67	25	8	18.9 (*p* < 0.001)
Which mask was the easiest to breathe through	12	46	42	7.13 (*p* = 0.028)
Which mask are you most likely to wear again?	67	21	12	18.4 (*p* < 0.001)

**Table 5:** Number (%) of children who removed their facemask whilst completing activities.

When Children Removed Their Mask	Vogmask	TuHao	ReSpimask
Whilst running only	7 (29)	6 (25)	15 (63)
Whist walking only	0 (0)	1 (4)	0 (0)
During both activities	2 (8)	2 (8)	0 (0)
Total	9 (38)	9 (38)	15 (63)

## References

[1] Holgate S.T. (2017). ‘Every breath we take: The lifelong impact of air pollution’—A call for action. Clin. Med..

[2] World Health Organization (WHO) (2016). Ambient Air Pollution: A Global Assessment of Exposure and Burden of Disease.

[3] World Health Organization (WHO) Ambient (Outdoor) Air Quality and Health, WHO. https://www.who.int/en/news-room/fact-sheets/detail/ambient-(outdoor)-air-quality-and-health.

[4] Royal College of Physicians, Royal College of Paediatrics and Child Health (2016). Every Breath We Take: The Lifelong Impact of Air Pollution.

[5] Walton H., Dajnak D., Evangelopoulos D., Fecht D. (2019). Impact Assessment of Air Pollution on Asthma in London.

[6] Zhang J., Mu Q. (2018). Air pollution and defensive expenditures: Evidence from particulate-filtering facemasks. J. Environ. Econ. Manag..

[7] Goh D.Y.T., Mun M.W., Lee W.L.J., Teoh O.H., Rajgor D.D. (2019). A randomised clinical trial to evaluate the safety, fit, comfort of a novel N95 mask in children. Sci. Rep..

[8] Greenhalgh T., Schmid M.B., Czypionka T., Bassler D., Gruer L. (2020). Face masks for the public during the covid-19 crisis. BMJ.

[9] Public Health England (2019). Review of Interventions to Improve Outdoor Air Quality and Public Health.

[10] Centers for Disease Control and Prevention (2014). Air Quality and Outdoor Activity Guidance for Schools.

[11] The National Personal Protection Technology Laboratory Respirator Fact Sheet: What You Should Know in Deciding Whether to Buy Escape Hoods, Gas Masks, or Other Respirators for Preparedness at Home and Work. https://www.cdc.gov/niosh/npptl/topics/respirators/factsheets/respfact.html.

[12] California’s Department of Public Health Wildfire Smoke FAQs. Respiratory Masks May be Beneficial for Some People. CDPH. https://www.cdph.ca.gov/Programs/EPO/Pages/BP_Wildfire_FAQs.aspx.

[13] McDonald F., Horwell C.J., Wecker R., Dominelli L., Loh M., Kamanyire R., Ugarte C. (2020). Facemask use for community protection from air pollution disasters: An ethical overview and framework to guide agency decision making. Int. J. Disaster Risk Reduct..

[14] Johnson A.T. (2016). Respirator masks protect health but impact performance: A review. J. Boil. Eng..

[15] Health and Safety Executive (2013). Respiratory Protective Equipment at Work: A Practical Guide.

[16] Cherrie J.W., Apsley A., Cowie H., Steinle S., Mueller W., Lin C., Horwell C.J., Sleeuwenhoek A., Loh M. (2018). Effectiveness of face masks used to protect Beijing residents against particulate air pollution. Occup. Environ. Med..

[17] Respilon Introduction, Czech Republic. https://www.respilon.com/AIR_EN.pdf.

[18] Willliams B.L. Sodium Chloride (NaCl) Aerosol Test Final Report.

[19] Vogmask, Premier Vogmask VMCV, Trees. https://www.vogmask.com/products/trees-vmcv.

[20] Galea K.S., Covey J., Timur S.M., Horwell C.J., Nugroho F., Mueller W. (2018). Short Communication: Health Interventions in Volcanic Eruptions—Community Wearability Assessment of Respiratory Protection against Volcanic Ash from Mt Sinabung, Indonesia. Int. J. Environ. Res. Public Health.

[21] Mueller W., Horwell C.J., Apsley A., Steinle S., McPherson S., Cherrie J.W., Galea K.S. (2018). The effectiveness of respiratory protection worn by communities to protect from volcanic ash inhalation. Part I: Filtration efficiency tests. Int. J. Hyg. Env. Health.

[22] Sandberg E.M. (1995). An evaluation of respiratory protective devices used in children’s evacuation. Ergonomics.

[23] Allison M.A., Guest-Warnick G., Nelson U., Pavia A.T., Srivastava R., Gesteland P.H., Rolfs R.T., Andersen S., Calame L., Young P. (2010). Feasibility of elementary school children’s use of hand gel and facemasks during influenza season. Influ. Other Respir. Viruses.

[24] TuHao N95 Particulate Respirator Masks with Valve, Child PM2.5 Dust Masks-Anti-Pollution Dustproof Cycling for Outdoor Safety Multi-Layer Protection. TuHao Kids Disposable Face Mask, Guangdong, China, B07KNB946R. https://www.amazon.co.uk/TuHao-Disposable-Particulate-Masks-Anti-Pollution-Multi-Layer/dp/B07KNB946R.

[25] IBM Corp (2019). Released. IBM SPSS Statistics for Windows, Version 24.0.

[26] The Weather Company London, United Kingdom Weather History. Weather Underground IBM. https://www.wunderground.com/history/daily/gb/london/EGLC/date/2019-7-7.

[27] Mauritzson-Sandberg E. (1991). Psychological effects on prolonged use of respiratory protective devices in children. Ergonomics.

[28] Covey J., Horwell C.J., Ogawa R., Baba T., Nishimura S., Hagino M., Merli C. (2020). Community perceptions of protective practices to prevent ash exposures around Sakurajima volcano, Japan. Int. J. Dis. Risk Reduct..

[29] King M.F., Bruner G.C. (2000). Social desirability bias: A neglected aspect of validity testing. Psychol. Market..

[30] Miller P.H., Baxter S.D., Royer J.A., Hitchcock D.B., Smith A.F., Collins K.L., Guinn C.H., Smith A.L., Puryear M.P., Vaadi K.K. (2015). Children’s Social Desirability: Effects of Test Assessment Mode. Pers. Individ. Differ..

[31] McCambridge J., Witton J., Elbourne D.R. (2013). Systematic review of the Hawthorne effect: New concepts are needed to study research participation effects. J. Clin. Epidemiol..

